# Mr. Ward's Cases of Inoculation for the Cow-Pox

**Published:** 1799-09

**Authors:** M. Ward

**Affiliations:** Manchester


### *To the Conductors of the Medical and Phyfical Journal.*

GENTLEMEN,

AS the difcuffion is not finally clofed, relative to the expediency of introducing the cow-pox into general practice, as a fubftitute for the fmall-pox, I fhall make no apology for tranfmitting to you the following cafes:—

It is a little extraordinary, that the firft patient whom I inoculated for the cow-pox, had a confluent eruption; and though the fymptoms were milder than I ever remember having feen them in the confluent fmall-pox, fhe was in fo much danger, as to make me very anxious about her from the 12th to the 21ft day.

During the continuance of the eruptive fever, fhe found relief from being carried out, and attended at the Infirmary, on my out-patient days, till the 1ft of May (the 15th day from the time fhe was inoculated, and the 4th day of the eruption), when the forenefs and debility became too confiderable to allow a continuance of the exercife. She then began to take opiates and acids, the falutary effects of which (efpecially of the opiates, repeated morning and evening), were very evident. Laxatives were occafionally neceffary, and the cerat. litharg. acetat. was applied for a few days towards the clofe of the diforder, to thofe parts where the difcharge was confiderable, to prevent her cloaths from adhering.

The fame vaccine matter was made ufe of in the firft nine cafes: the other five patients were inoculated with matter taken from MARTHA MEAD.

MANCHESTER, *July* 12, 1799

M. WARD.

*Firft Cafe.*

April 16, 1799. Martha Mead, aged feven years. Fourth day. The punctures are confiderably inflamed; the fuperior one difcharges a little matter. Eighth day, there is lefs efflorefcence round the punctures than on the fourth fourth day. Thirteenth day, the fuperior punćture is healed, the inferior one has the appearance of an oblong veficle, and contains a limpid fluid; it is more elevated than on the 8th day; there is alfo a greater degree of efflorefcence. She began to be feverifh yefterday morning, and continued fo all day; had a reftlefs night, and was delirious; but walked to the Infirmary this morning, and is relieved by being out; her breath is offenfive, tongue white, appetite bad, and her eyes look dull and heavy. Fifteenth day, fhe flept tolerably the laft night, vomited three times the night before, and was very ill yefterday till noon, when fome fpots were obferved on her face, which relieved her. Towards evening a great number of puftules appeared on her neck, breaft, and arms; and this morning there is a plentiful crop on every part of her body. Her arm is more inflamed than on the thirteenth day; her tongue is white and foul; thirft great; her appetite bad; but fhe flept better laft night than fhe has done fince the eruptive fever attacked her.

Sixteenth day. She has had a reftlefs night, and complains of her throat being fore. Several puftules are vifible on the tonfils and mucous membrane of the throat; in other refpećts fhe is much the fame as yefterday The eruption is now confluent in fome places on her face and arms.

Seventeenth day. She flept well, and is not fo feverifh; but fhe is rather hoarfe, and very dull, and complains of general forenefs.

Eighteenth day. She has flept well; is very weak; throat fore.

Nineteenth day. She has not had a good night; her tongue is foul and very fore; there are feveral puftules upon it; throat fomewhat better; her eye-lids are much fwelled, and nearly clofed; the puftules become more confluent daily; fhe is very unwilling to be moved.

Twentieth day. The patient has had a reftlefs night, and complains heavily of the forenefs; her throat is better; her eye-lids are clofed; the puftules continue to run more into each other; maturation feems nearly complete every where but on the legs and feet; the inoculated arm is painful from the fhoulder to the elbow; it is much loaded with puftules; the puncture is covered with a fmall dry fcab.

Twenty-firft day. She has been reftlefs, but is better this morning; her throat is well, and the hoarfenefs gone; the forenefs is fo great that fhe moans and cries continually, except when quieted by opiates.

Twenty-fecond day. She has flept well and is much better; fhe can open her eyes with eafe. Many of the puftules have dropped off from her face fince yefterday, and have left the fkin of a deep red colour, but not pitted.

The

The child has complained several days of pain in her left knee, a cluster of pustules having joined so as to form one very large one. It broke last night and she is easier.

Twenty-third day. She has slept well, and makes no complaint, except of soreness on the bottoms of her feet, which prevent her from walking. It is surprising with what rapidity the pustules fall off from her breast and neck, leaving only a redness and a roughness on the skin.

Twenty-fourth day. She is going on well in every respect. The pustules continue to exfoliate rapidly.

Twenty-sixth day. She has had an uneasy night; there is a considerable discharge from the pustules on her sides, back, and belly.

Twenty-eighth day. Has rested well the two last nights, but is very tedious and uneasy to day; she complains that "her cloaths stick to her body like bird-lime."

Thirty-fifth day. Her health is restored.

July 4. She was inoculated with variolous matter.

July 11. She has not taken the infection.

I have only to add to the above statement, that the eruptive fever was not so strongly marked by debility, as it usually is in the confluent small-pox: the secondary fever was so trifling as to be scarcely worth mentioning; the progress of the pustules to maturation, as well as their subsequent separation, was more rapid, the separation was also more complete, *and the exhalations were all along free from any unpleasant odour*. As nearly as I could ascertain, she had not fewer than from 1600 to 1800 pustules.

Though she lives in a populous part of the town, I have reason to believe the disease has not been communicated from her to any other person in the neighbourhood, having taken some pains to gain information on this point.

*Second Case.*

April 16, 1799. WILLIAM MEAD, aged nine months. He was attacked with feverish symptoms on the eighth day, at which time some elevation of the punctures, accompanied by some degree of efflorescence, had taken place. These appearances decreased from that time to the eleventh day, when they were quite gone, and on the fifteenth day one of the punctures was healed, and the other was covered with a small dry crust. The child continued well from the eleventh to the thirty-second day. A slight ulceration began to take place under the scab on the twenty-first day, which gradually dually increased to the size of a silver three-pence, but it was so superficial, I did not think it necessary to apply any thing; a little ichor was discharged from the place from this time to the thirty-second day, when he became feverish, and continued very restless and tedious three or four days. On the thirty-third, thirty-fourth, and thirty-fifth days, about fifty pustules appeared, most of which were on his face. The discharge ceased, and his arm got well, soon after the fever abated.

I was unable to account for the fever coming on so late as the thirty-second day (and it seemed a strong argument against introducing the cow-pox into general practice) till I had read Dr. JENNER's second publication on the *Variolæ Vaccinæ*. He there attributes this circumstance, in some instances which he has met with, to the secondary effects of the virus, and says he is able to prevent them, by applying escharotics to the part affected.

July 4. I inoculated him with variolous matter.

July 11. The punctures are healed.

*Third Case.*

April 16, 1799. SARAH GRAY, aged five months, was inoculated.

April 23. She was inoculated again, the matter inserted on the 16th having produced no effect. Fourth day:—She has not taken the infection; variolous matter was inserted. Eighth day:—The eruptive fever has taken place. Twelfth day:—She has a fine, distinct sort.

*Fourth and Fifth Cases.*

April 16, 1799. ANN MORLEY, aged five years, and MARY ANN MORLEY, aged nine months. Eighth day:—They have not either of them taken the infection, and were therefore inoculated a second time, and with the same event. April 30:—They were inoculated with variolous matter, but without success, and they were inoculated again with variolous matter on the 5th of May. Eighth day:—The punctures are a little elevated and inflamed. Seventeenth day:—The children have not been indisposed since the last inoculation, nor have any pustules appeared on either of them. The mother is certain they have not had the small-pox, and they were in every respect healthy each time of their being inoculated.

*Sixth Case.*

April 16, 1799. GEORGE WOOD, aged three years, was inoculated with vaccine matter, and afterwards with variolous matter, but without effect in both instances; but it must be observed, he had been inoculated four months before, and that his arm was inflamed at that time; a trifling indisposition also took place, but no pustules appeared.

*Seventh Case.*

April 19, 1799. Thomas Coop, aged five months. April 23:—He was inoculated again, the first inoculation having failed. April 26:—The punctures are healed. He was inoculated with variolous matter. Eighth day:—The inflammation on his arm is pretty considerable. Fifteenth day:—The child has not been indisposed, nor had any pustules. His arm is well.

*Eighth Case.*

April 19, 1799. Samuel Barnes, aged sixteen months. April 23:—Not having taken the infection, he was inoculated again. Eighth day:—There appears about as much inflammation round the incisions as usually takes place on the third or fourth day, when infection has been produced by variolous matter. He has been hot and feverish since the fifth day, owing to his teeth, as his mother supposes, and this opinion is strengthened by the state of the arm. Eleventh day:—There is a good deal of efflorescence round the punctures; he was restless last night, and is tedious and hot to-day. Eighteenth day:—He is very well and has not had any pustules. Twenty-ninth day:—I inoculated him with variolous matter in both arms; but no disease ensued.

*Ninth Case.*

April 23, 1799. James Hopwood, aged nineteen weeks. The punctures being healed on the 26th, he was inoculated again with vaccine matter. Fifth day:—His arm is inflamed from the vaccine matter inserted four days since. He was feverish yesterday, but is better to-day. His mother thinks he is about his teeth, but his gums are not swelled. Eighth day. The inflammation advances on his arm; he looks pale, and is hot and restless. Twelfth day:—No complaint; the punctures are healed; he has not had any pustules, except a few on the inoculated arm, close to the incisions. I this day inoculated him with variolous matter. Nineteenth day:—He has taken the infection. Twenty-second day. He has a mild disease, and thirty pustules.

*Tenth Case.*

April 30, 1799. John Lunt, aged fourteen weeks. He took the infection, and sickened on the seventh day. He began to break out on the tenth, and was pretty full of pustules on the twelfth, but the eruption was distinct. There was an unusual number of white pustules, as well as a great degree of efflorescence round the punctures, on the eleventh day; and the pustules being placed at regular distances from each other, the arm assumed a studded appearance. He continued to be more or less feverish from the seventh day to the eighteenth, and was uncommonly tedious; indeed he was seldom feldom quiet during that time, except when he was under the influence of tincture of opium, or when he was carried out. On the laft mentioned day his eye-lids were fwelled and inflamed; thefe appearances continued feveral days. A hoarfenefs came on the eleventh day, and did not leave him till the eighteenth. On the twenty-fecond day, however, his health was completely reftored, and on the twenty-fourth he was inoculated with variolous matter, but without effect.

*Eleventh Cafe.*

William Bullock, aged nine months. He took the infection, and broke out on the tenth, eleventh, and twelfth days, and had about fixty puftules, befides a great number in the neighbourhood of the punctures; he was poorly three days before they came out, and one after. He was fubfequently inoculated with variolous matter, but without effect.

*Twelfth Cafe.*

Robert Foley, aged fix months. As he did not take the infection, I inoculated him again in ten days, but his mother neglected to bring him in and I have not heard of him fince.

*Thirteenth and Fourteenth Cafes.*

Henry Delmore, aged three years, and Elizabeth Delmore, aged fix months. Their place of refidence was not noted, and I have not heard of them fince.

The great queftion relative to the cow-pox, feems now to be brought within a narrow compafs.

We have the authority of a phyfician whofe experience in the inoculated cow-pox is very extenfive, for afferting, that thofe perfons who have had this difeafe by inoculation, are thereby fecured from having the fmall-pox. *

The fame gentleman has lately found, that the inoculated cow-pox is feldom accompanied by puftules, if the virus be taken from thofe perfons who have had the difeafe in its mildeft form †.

Confidering the above obfervations as eftablifhed facts, and fhould it alfo appear, that the cow-pox is not fo liable to be propagated by contagion, as the fmall-pox; may we not indulge a hope, that the æra is probably not far diftant, when we fhall be able to congratulate mankind at large, on their having a fair profpect of being exempted, at no very remote period, from that moft deftructive malady.

To

* See "Woodville's Cases and Remarks on the Cow-pox."

† See the "Medical and Physical Journal for July, No. V."

